# CDKL5 regulates the initiation of retrograde axonal transport through CLIP170–dynactin complex formation

**DOI:** 10.1111/febs.70230

**Published:** 2025-08-23

**Authors:** Serena Baldin, Clara Carmone, Giorgia Valetti, Roberta De Rosa, Isabella Barbiero

**Affiliations:** ^1^ Department of Biotechnology and Life Sciences, Centre of NeuroScience University of Insubria Busto Arsizio Italy

**Keywords:** CDKL5, microtubules, neurodevelopmental disorder, neurons, transport

## Abstract

Cyclin‐dependent kinase‐like 5 (CDKL5) is a serine–threonine kinase implicated in regulating microtubule (MT) dynamics. Mutations in CDKL5 are associated with a rare neurodevelopmental disease called CDKL5 deficiency disorder (CDD), which is characterized by early‐onset seizures and intellectual disabilities. Microtubule (MT)‐related functions of CDKL5 are in part correlated with its interaction with MT‐associated proteins, such as CAP‐Gly domain‐containing linker protein 1 [CLIP1; also known as cytoplasmic linker protein 170 alpha‐2 (CLIP170)]. CLIP170 is a MT plus‐end tracking protein that, once activated, can bind MTs and other proteins, favoring MT dynamics. Importantly, we have previously shown that CLIP170 is inactive in the absence of CDKL5, thus hindering MT functions. One of the best‐characterized interactors of CLIP170 is dynactin, a multisubunit complex that binds the motor protein dynein. In particular, in neurons, active CLIP170 localizes to MTs in the axonal periphery, where it serves as a docking site for the interaction with dynactin, which in turn recruits dynein and various cargos, favoring the initiation of retrograde transport toward the neuronal soma. Here, we demonstrated that CLIP170–dynactin complex formation is impaired in the absence of CDKL5, thus leading to defective retrograde cargo trafficking. Overall, our findings expand the knowledge on the molecular mechanisms underlying neuronal transport and provide novel information regarding the etiopathogenesis of CDD.

## Graphical abstract

Activation of CLIP170 by CDKL5 in wild‐type (WT) cells promotes its open conformation and association with tyrosinated microtubule (MT) plus‐ends (+), where it works as a docking site to recruit the dynactin–dynein–cargo complex, promoting the initiation of retrograde transport. In knock‐out (KO) neurons, CLIP170 is mainly present in a closed (inactive) conformation that impedes its MT‐binding and recruitment of the dynactin–dynein complex, hindering retrograde transport initiation.

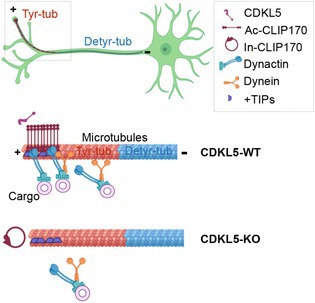

**Abbreviations**
CDD: CDKL5 deficiency disorder
WT: wild‐type
KO: knock out
+TIPs: plus‐end tracking proteins
MTs: microtubules
DIV: days *in vitro*
PREG: pregnenolone
IP: immunoprecipitation
IF: immunofluorescence
WB: western blot

## Introduction

CDKL5 deficiency disorder (CDD) is an X‐linked neurodevelopmental encephalopathy caused by mutations in the *CDKL5* (cyclin‐dependent kinase‐like 5) gene. Refractory epilepsy starting within three months after birth, cognitive and motor developmental delays represent together with gastrointestinal problems, sleep, and breathing disturbances the common diagnostic criteria to identify CDD patients [1]. CDKL5 is a serine–threonine kinase that is highly abundant in the brain where it is largely expressed in neuronal nuclei, axons, dendrites, and dendritic spines [2]. Loss of CDKL5 both *in vivo* and *in vitro* is associated with morphological defects, resulting in global neuronal atrophy characterized by reduction in axon length, dendritic complexity, and depletion of mature spines [3, 4, 5, 6, 7]. The emerging role of CDKL5 in the regulation of microtubule (MT) dynamics can at least in part explain these defects [8].

MTs are long polarized polymers formed by dimers of α and *β* tubulin, the primary function of which in all cell types is to act as railways for the transport of cargo throughout the cytoplasm; this property allows the sorting of basic building blocks such as proteins, lipids, mitochondria, and synaptic vesicles in axons and dendrites and is fundamental to coordinate the morphological changes that occur during neuronal maturation [9]. Not surprisingly, defective MT‐dependent transport is detrimental to neurons and is implicated in neurodegenerative and neurodevelopmental disorders [9]. In the axon, the growing MT plus‐ends (terminated by *β*‐tubulin) are oriented toward the axon tip. This well‐established polarized organization provides the tracks for the transport of cargos that are mediated by two classes of motor proteins, dynein and kinesin, which move toward the minus‐ (retrograde transport) and plus‐ends (anterograde transport), respectively. Dynein functions, in particular, are highly regulated by activating proteins such as dynactin, a multisubunit complex that enhances the processivity of dynein and mediates cargo interaction [10, 11].

Motor‐cargo recruitment to MTs is one of the first steps in neuronal transport initiation; recent evidence suggests that this process is influenced by tubulin post‐translational modifications (PTMs) and plus‐end tracking proteins (+TIP) [12]. In this regard, we have recently demonstrated that CDKL5 regulates the functioning of CLIP170, a key +TIP that is implicated in regulating MT dynamics [13]. CLIP170 was the first +TIP to be discovered [14]. It is characterized by an N‐terminal portion that includes the Cap‐Gly domain (the MT localizing motif) surrounded by serine‐rich regions, and a long C‐terminal coiled‐coil tail with two metal‐binding domains (zink fingers) [15]. CLIP170 switches from a closed‐inactive conformation resulting from the interaction between the Cap‐Gly domain and the zinc knuckles to an open‐active conformation that allows the protein to bind MT plus ends, promoting their elongation [15]. The open‐active conformation has turned out to be functional also for the interaction of CLIP170 with other binding partners such as the p150^glued^, which represents the largest subunit of dynactin [16].

At the neuronal level, CLIP170 was demonstrated to promote dendritogenesis [17] and the development of neuronal precursors into polarized mature neurons [18]. In particular, it is enriched in the axonal growth cone where it stabilizes the MT plus‐ends by allowing them to protrude into the actin zone, thus promoting the elongation of the axon [18]. The correct binding of CLIP170 to dynamic MTs in the growth cone was further demonstrated to allow the recruitment of p150^Glued^, which in turn, as mentioned, activates dynein leading to the initiation of retrograde transport [19].

Importantly, the association of CLIP170 to MTs depends on the presence of CDKL5; indeed, we recently found that the absence of CDKL5 in primary hippocampal neurons causes the delocalization of CLIP170 from MTs within the growth cone [3]. In line with the role of CDKL5 in regulating CLIP170 functioning, we found that in *Cdkl5*‐silenced cells CLIP170 is mostly present in a closed‐inactive conformation that reduces its affinity for MTs and for its binding partner IQGAP1, a scaffold protein implicated in the stabilization of MTs at the cellular cortex of polarized cells [13]. Importantly, CLIP170 was demonstrated to be a direct interactor of the neuroactive steroid pregnenolone (PREG), which promotes the open‐active conformation of the protein, thus favoring its binding with MTs and proteins, including p150^glued^ [16]. Accordingly, we found that PREG is capable of restoring the MT association of CLIP170 and defects in growth cone morphology in *Cdkl5*‐KO neurons [3].

The CDKL5‐dependent hypofunctionality of CLIP170 suggests a possible implication of the kinase in motor‐cargo recruitment: a mechanism never studied before that in part may explain the delay in neuronal maturation and the increase in death susceptibility that has been described in *Cdkl5‐*KO neurons [20].

Here, we used biochemical and live imaging approaches to elucidate the role of CDKL5 in the initiation of retrograde transport at the axonal growth cone. Our data show that CDKL5 is required for the correct localization of dynactin at the plus‐ends of MTs and its distal enrichment in the axon, leading to an efficient flux of cargo from the distal neurite. We further found that the mislocalization of dynactin from MT tips, observed in the absence of CDKL5, corresponded to a reduction in its interaction with CLIP170. Finally, we found that the treatment with PREG restored the CLIP170‐p150^glued^ interaction and transport efficiency along the axon in *Cdkl5‐*KO neurons. Overall, these findings highlight a new role of CDKL5 in the regulation of axonal transport, broadening the knowledge of the etiopathogenesis of CDD.

## Results

### Loss of CDKL5 alters the distribution of p150^glued^ at the MT‐tips

**Fig. 1:**
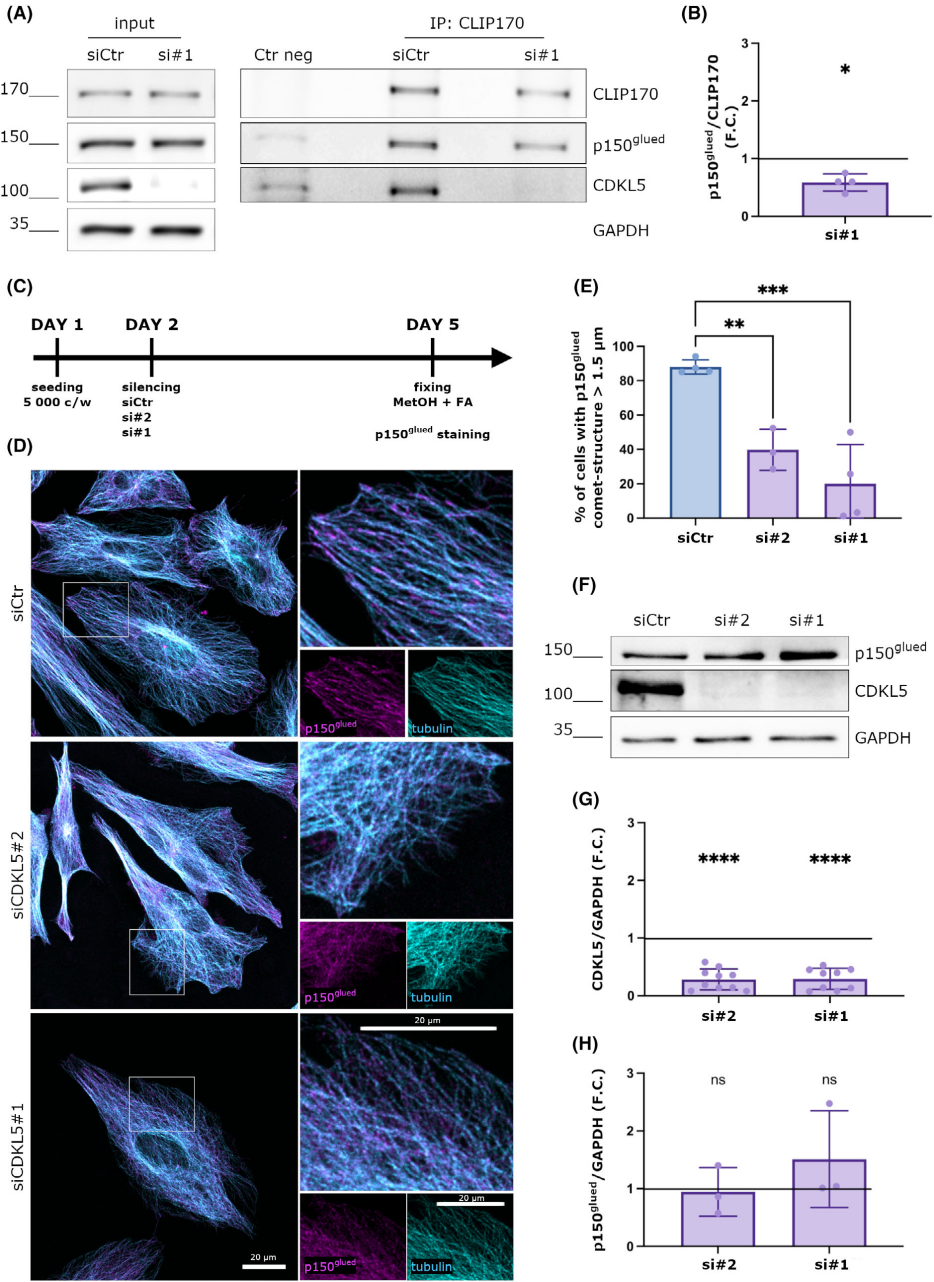
Loss of CDKL5 alters the distribution of p150^glued^ at the MT‐tips. (A) HeLa cells were silenced with a control siRNA (siCtr) and a siRNA against *CDKL5* (siCDKL5#1) for 72 h; CLIP170 was immunoprecipitated from whole‐cell lysates using IgGs as a negative control. Inputs and immunoprecipitates were analyzed by western blotting with the indicated antibodies. (B) Graph showing the relative amount of p150^glued^ that co‐precipitates with CLIP170 expressed as a fold change (F.C.) of the siCtr condition. Data are presented as mean ± SD (*P < 0.05), one‐sample *t* test (*n* = 4). (C) Timeline of cell treatment. (D) Representative image of HeLa cells silenced with siCtr, siCDKL5#1, or si*CDKL5*#2 for 72 h and stained with a p150^glued^ antibody in magenta and alpha‐tubulin antibody in cyan. In control cells, p150^glued^ localizes on microtubules forming comet‐like structures, whereas diffuse staining is present in silenced cells. Scale bar 20 μm. Image orientation has been adjusted to better visualize the comet‐like structures. (E) Graph showing the percentage of cells with p150^glued^ comet structures whose length is > 1.5 μm. Data are shown as mean ± SD (**P < 0.01, ***P < 0.001), one‐way ANOVA, Dunnett's *post hoc* test (siCtr = 237 cells, si*CDKL5*#1 = 261 cells, si*CDKL5*#2 = 117 cells were analyzed; from 4 different experiments for siCtr and si*CDKL5*#1 and from different experiments 3 for si*CDKL5*#2). (F) Representative western blot showing p150^glued^ and CDKL5 levels in control and silenced cells. GAPDH was used as a loading control. (G) Graph showing the CDKL5 expression levels normalized with GAPDH, expressed as a fold change (F.C.) of control cells. Data are shown as mean ± SD (****P < 0.0001), one‐sample t test (*n* = 9 for si*CDKL5*#1 and *n* = 10 for si*CDKL5*#2). (H) Graph showing the quantification of p150^glued^ levels in CDKL5 silenced cells, normalized with GAPDH, expressed as a fold change (F.C.) of control cells. Data are shown as mean ± SD (ns), one‐sample *t* test (*n* = 3).

The silencing of CLIP170 in HeLa cells causes a more diffuse localization of the dynactin subunit p150^glued^ with respect to the MT‐tips [15]. Considering that CLIP170 is mainly present in a closed‐inactive conformation in CDKL5 deficient cells [21], we hypothesized that CDKL5 loss might impact the interaction between CLIP170 and p150^glued^. To this purpose, we used a CDKL5‐targeting siRNA to reduce CDKL5 expression in HeLa cells, while a nontargeting siRNA served as a negative control. Following this, CLIP170 was immunoprecipitated from the lysates to assess the amount of associated p150glued. Western blotting (WB) was first performed on input lysates to evaluate the efficiency of CDKL5 knockdown and to examine the levels of CLIP170 and p150glued. As shown in Fig. 1A, CDKL5 expression was efficiently reduced without affecting the expression levels of CLIP170 or p150glued. Additionally, WB analysis of the immunoprecipitated complexes confirmed the successful enrichment of CLIP170 in both control and CDKL5‐silenced conditions. However, the quantity of p150glued that co‐immunoprecipitated with CLIP170 was notably decreased in the absence of CDKL5, with a reduction of about 40% compared to the control (Fig. 1B). Considering that CLIP170 is fundamental to promote the correct localization of p150^glued^ at the MT‐tips [15], we speculated that the absence of CDKL5, by interfering with CLIP170 functionality and p150^glued^ association, could have a negative effect on p150^glued^ accumulation at the MT‐tips. To address this point, we silenced CDKL5 expression in HeLa cells for 72 h with two different siRNAs against *CDKL5* (si*CDKL5*#1 and #2. Fig. 1C) and analyzed through immunofluorescence the localization of p150^glued^ at the MT‐tips (Fig. 1D). As shown in Fig. 1D, in control cells, p150^glued^ accumulate correctly at the MT‐tips where it forms comet‐like structures. However, in cells treated with *CDKL5* siRNAs, a much more diffuse pattern of p150^glued^ was observed (Fig. 1D). In particular, by analyzing the percentage of p150^glued^ comet‐like structures presenting a length greater than 1.5 μm, which represents the reference length of p150^glued^ positive comets in fixed cells [22], we found that p150^glued^ formed comet‐like structures in almost 90% of analyzed siCtr cells. Conversely, in the absence of CDKL5, p150^glued^ accumulated in comet‐like structures only in 20% of siCDKL5#1 cells and in 40% of siCDKL5#2 cells (Fig. 1E). We further confirmed the efficiency of silencing by WB (Fig. 1F); indeed, the two different siRNAs produced a reduction in CDKL5 expression of 70% with respect to the control cells (Fig. 1G). As above, in CDKL5‐silenced cells, we did not observe any change in the total expression levels of p150^glued^ (Fig. 1H), thus suggesting that the diffuse pattern of dynactin is caused by a functional alteration of the protein.

### CDKL5 overexpression restores p150^glued^ comets at the MT‐tips

**Fig. 2:**
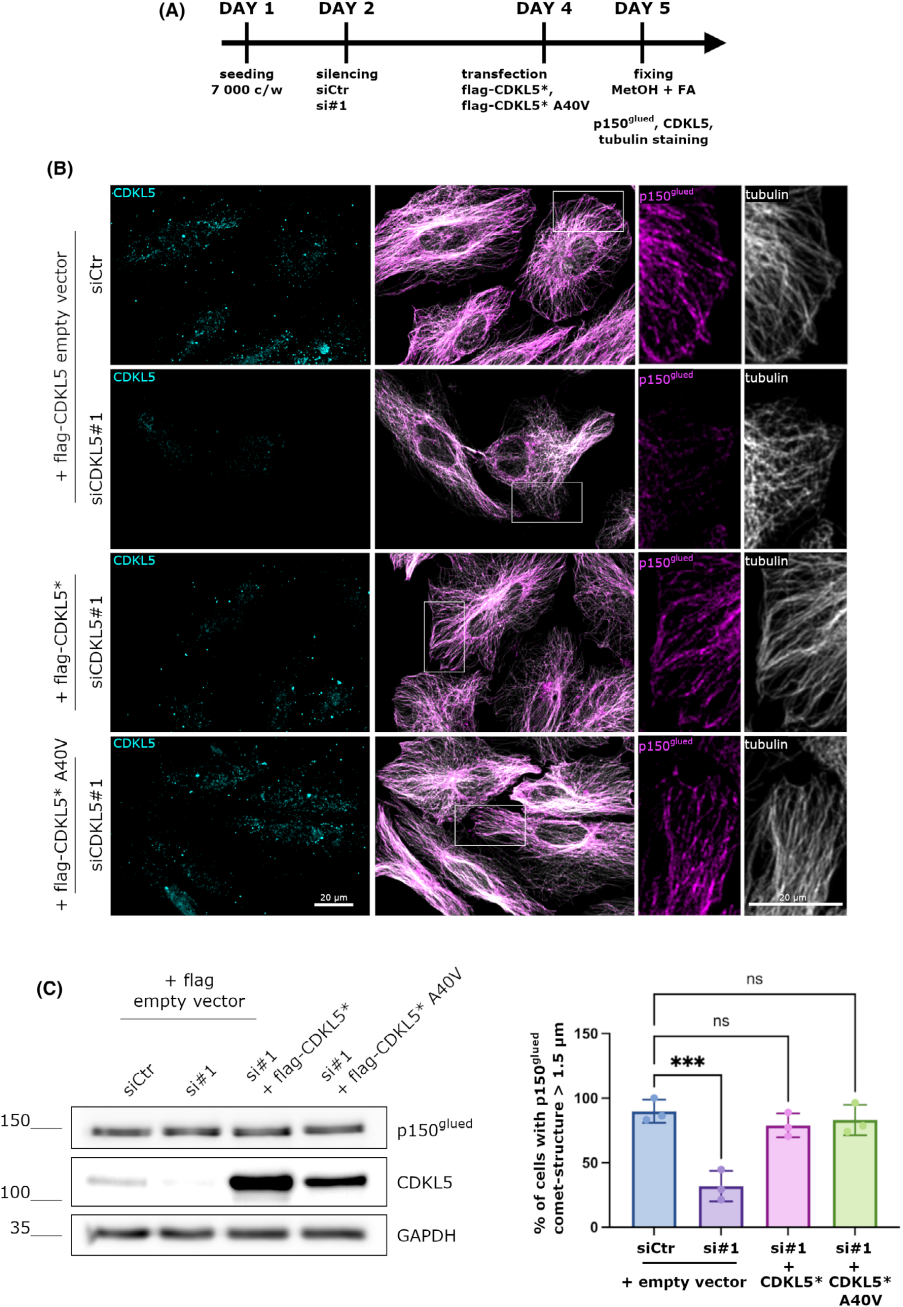
CDKL5 overexpression restores p150^glued^ comets at the MT‐tips. (A) Timeline of cell treatment. (B) Representative image of HeLa cells silenced with control siRNA (siCtr) and si*CDKL5*#1 for 48 h and transfected with a construct expressing a siRNA resistant CDKL5 (CDKL5*) or a siRNA resistant CDKL5 deprived of its catalytic activity (CDKL5* A40V) together with a flag empty vector alone for 24 h. Cells were stained with CDKL5 antibody in cyan to verify the correct silencing and transfection, p150^glued^ antibody in magenta, and alpha‐tubulin antibody in white, respectively. Scale bar 20 μm. Image orientation has been adjusted to better visualize the comet‐like structures. (C) Representative western blot showing p150^glued^ and CDKL5 levels in control and siCDKL5#1 silenced cells upon flag empty vector and flag‐CDKL5* or flag‐CDKL5* A40V vectors transfection. GAPDH was used as loading control. (D) Graph showing the percentage of cells with p150^glued^ comet‐structures whose length is > 1.5 μm. Data are shown as mean ± SD (***P < 0.0001); one‐way ANOVA, Dunnett's *post hoc* (109 and 100 respectively of siCtr and si*CDKL5*#1 plus flag empty vector transfected cells and 66 and 69 siCDKL5#1 cells transfected with flag‐CDKL5* or flag‐CDKL5* A40V vector respectively; from 3 different experiments).

To deeply investigate the involvement of CDKL5 in controlling the correct localization of p150^glued^, we performed a rescue experiment in HeLa cells by re‐expressing CDKL5. To this aim, HeLa cells were silenced for 48 h with control and *CDKL5*#1 siRNAs and then transfected with a construct expressing a si*CDKL5*#1‐resistant CDKL5 derivative (CDKL5*) for 24 h (Fig. 2A,B). As expected, CDKL5, which exhibited a clearly dot‐like pattern well‐visible both in the nucleus and in the cytoplasm [23, 24], was efficiently silenced in the siCDKL5#1 treated cells and correctly overexpressed in the CDKL5* expressing cells (Fig. 2B). The silencing and overexpression efficiency was further evaluated by WB (Fig. 2C). As expected, p150^glued^ formed comet‐like structures at the MT plus‐ends of control cells; conversely, the absence of CDKL5 was associated with a more diffuse pattern of the protein (Fig. 2B). Importantly, the transfection of the siRNA resistant CDKL5 derivative allowed p150^glued^ to localize correctly at the MT plus‐ends, restoring the situation of the control cells (Fig. 2B). Importantly, we obtained an analogous effect following the overexpression of the kinase‐dead derivative of CDKL5 (CDKL5‐A40V). Specifically, we observed that 89% of control cells presented p150^glued^ comet‐like structures with a length greater than 1.5 μm. The percentage was reduced to 31% upon CDKL5 silencing, whereas the re‐expression of both CDKL5‐WT and CDKL5‐A40V restored the localization of p150^glued^ to 78% and 83% of cells, respectively (Fig. 2D). We further found that the re‐expression of CDKL5 WT and CDKL5‐A40V did not impair the expression levels of p150^glued^ as shown by the WB (Fig. 2C).

### The absence of CDKL5 interferes with the interaction of CLIP170 and p150^glued^ at the MT‐tips

Considering the above results, we wanted to analyze whether the delocalization of the p150^glued^ in CDKL5 deficient cells reflects a reduction in its association with CLIP170. However, the large number of MT‐tips made a precise quantification of the p150^glued^–CLIP170 accumulation on MT‐tips in HeLa cells difficult. Therefore, we changed to COS7 cells, which have a more scattered MT cytoskeleton.

**Fig. 3:**
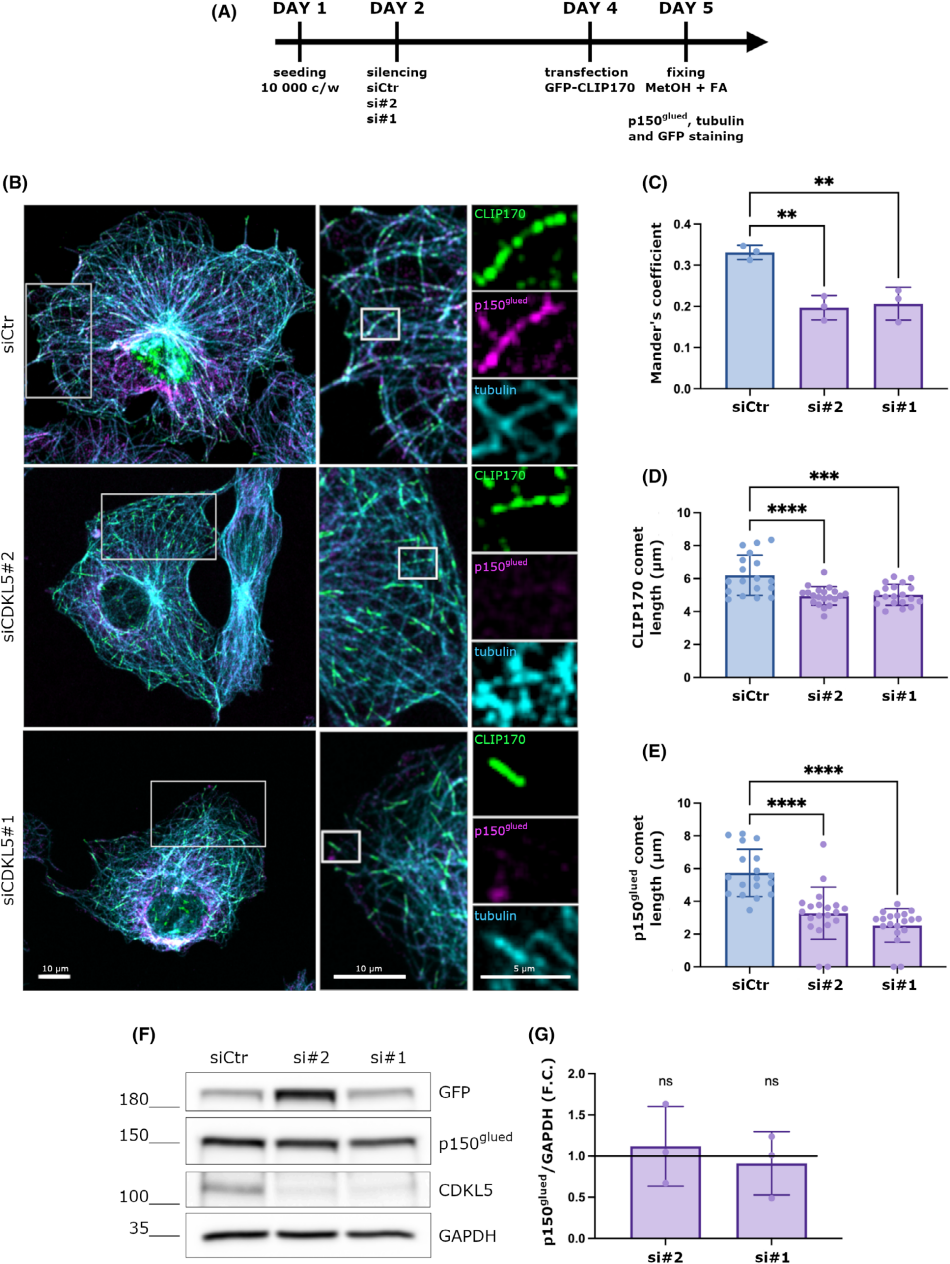
The absence of CDKL5 interferes with the interaction of CLIP170 and p150^glued^ at the MT‐tips. (A) Timeline of the treatment (B) Representative image of COS7 cells silenced with control siRNA (siCtr), si*CDKL5#*1, or si*CDKL5*#2 for 48 h and transfected with a GFP‐CLIP170 construct for 24 h. Cells were stained with a p150^glued^ antibody in magenta, alpha‐tubulin antibody in cyan, and ɑ‐GFP. As shown, in control cells, p150^glued^ colocalizes with CLIP170 at the MT tips, forming comet‐like structures. In CDKL5 silenced cells, the signal of p150^glued^ is diffuse. Scale bar 10 μm. Image orientation has been adjusted to better visualize the comet‐like structures. (C) Graph showing the colocalization between CLIP170 and p150^glued^ comets expressed as a Mander's coefficient. This is reduced in CDKL5 silenced cells. Data are shown as mean ± SD (**P < 0.001), one‐way ANOVA, Dunnett's post hoc (siCtr = 17cells, si*CDKL5*#1 = 15 cells, si*CDKL5*#2 = 17 cells, from 3 different experiments). (D, E) Graphs showing the length of CLIP170 (D) and p150^glued^ (E) fluorescent signals (“comet‐like” structures) at the MT plus‐end. Data are presented as mean ± SD, ****P < 0.0001, ***P < 0.001), one‐way ANOVA followed by Dunnett's post hoc test (siCtr = 18 cells, siCDKL5#2 = 20 cells, siCDKL5#1 = 19 cells were analyzed from 3 different experiments). (F) Representative western blot that verifies the efficient silencing of CDKL5 and the transfection with the GFP‐CLIP170 construct. GAPDH was used as a loading control. (G) Graph showing the quantification of p150^glued^ levels in CDKL5 silenced cells, normalized with GAPDH, expressed as a fold change (F.C.) of control cells. Data are shown as mean ± SD (ns), one sample *t*‐test (*n* = 3).

COS7 cells were thus silenced for 48 h with a Ctr and two different CDKL5 siRNAs and transfected with a GFP‐CLIP170 expressing vector. After 24 h, cells were fixed with methanol and formaldehyde and stained for GFP to enhance the CLIP170 signal, alpha‐tubulin, and p150^glued^ (Fig. 3A,B). Of note, exogenously expressed GFP‐CLIP170 was verified in the past to faithfully bind MTs like the endogenous protein [25]. The colocalization rate was calculated using the Mander's coefficient as shown in Fig. 3C; the absence of CDKL5 significantly impaired the colocalization of p150^glued^ and CLIP170 at the tips of MTs (Fig. 3C).

We further analyzed the length of both CLIP170 and p150^glued^ comet‐like structures. In accordance with previous data, the accumulation of CLIP170 on MT‐tips was compromised in CDKL5‐silenced cells as shown by shorter comets at the MT‐tips in comparison with control cells (Fig. 3D) [13]. Interestingly, the same impairment was also observed for p150^glued^ (Fig. 3E). This suggests that the loss of CDKL5 hampers the ability of CLIP170 to productively bind MTs and that the reduced CLIP170 binding impacts the recruitment of dynactin.

We finally verified whether the reduced p150^glued^ signal at the MT‐tips was caused by an overall reduction of its expression levels in CDKL5‐silenced COS7 cells. Through a WB analysis, we confirmed a strong silencing of CDKL5 but no effect on p150^glued^ levels (Fig. 3 F, G). This confirmed that the impaired accumulation of dynactin on MT ends is not due to reduced expression levels but rather a consequence of CLIP170 hypo‐functionality.

### CDKL5 influences the distal localization of p150^glued^ in neurons

**Fig. 4:**
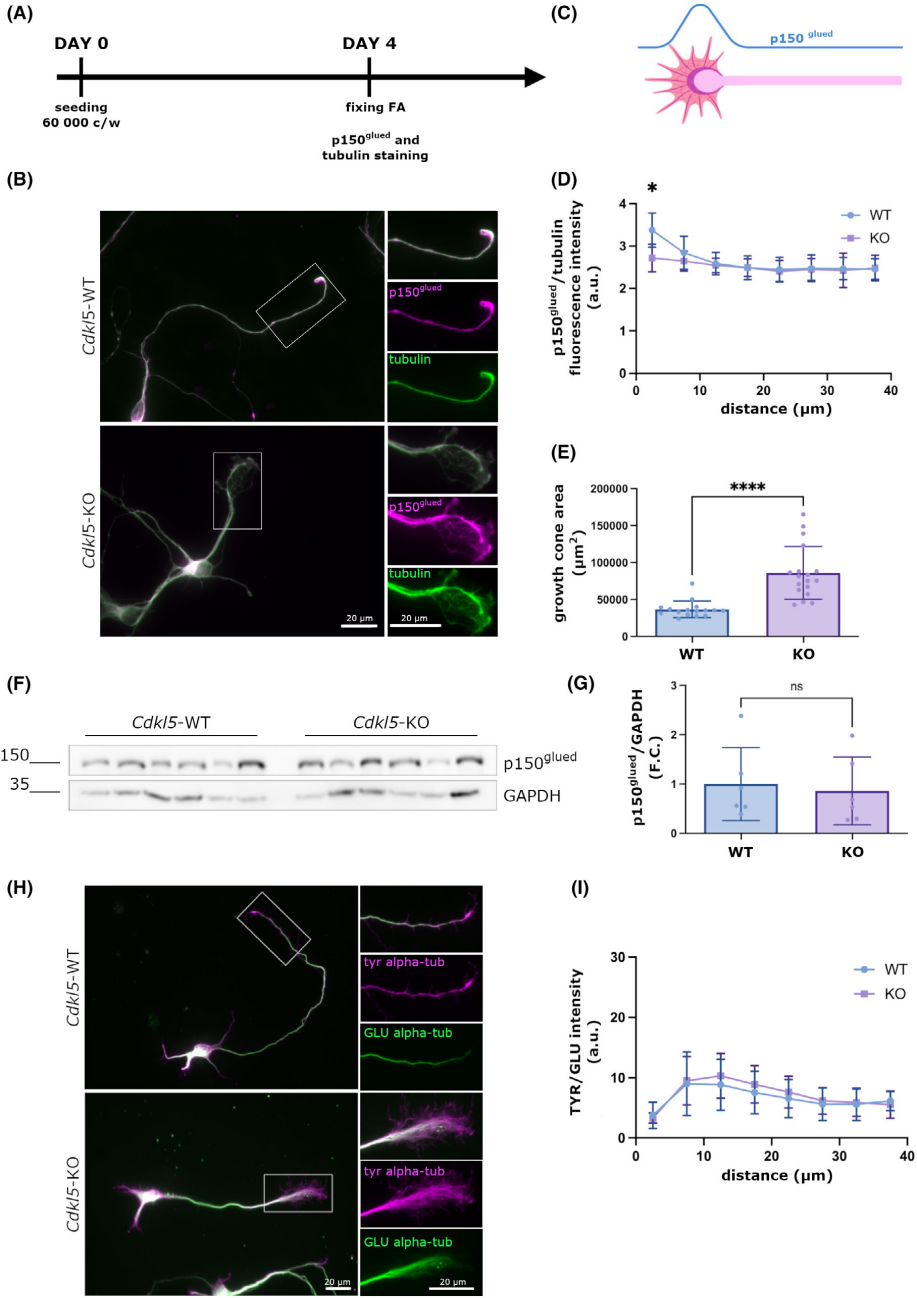
CDKL5 influences the distal localization of p150^glued^ in neurons. (A) Timeline representing preparation (DIV0) and analysis (DIV4) of neuronal cultures. (B) Representative image of *Cdkl5*‐wild‐type (WT) and knockout (KO) neurons (DIV 4) stained with antibodies against p150^glued^ (magenta) and tubulin (green). Scale bar 20 μm. Image orientation has been adjusted to facilitate direct comparison between the morphology of WT and KO growth cones. (C) The figure shows a schematic representation of p150^glued^ line scan analysis: a segmented line of 40 μm was drawn following the tubulin signal, starting from the tip of the growth cone and a plot profile of p150^glued^ and tubulin was obtained. (D) Graph showing the ratio of p150^glued^/alpha‐tubulin fluorescence intensity in *Cdkl5*‐KO and WT distal axon (40 μm line intensity). Data are presented as mean ± SD, (*P < 0.05), unpaired multiple *t*‐test (16 *Cdkl5*‐WT and 18 *Cdkl5‐*KO neurons were analyzed). (E) Graph showing the growth cone area quantification of the same neurons. Data are presented as mean ± SD, (****P < 0.0001), unpaired *t*‐test. (F, G). Representative western blot and the corresponding graph showing the unchanged p150^glued^ levels of expression in *Cdkl5*‐WT and KO neurons (DIV 4). GAPDH was used as loading control (*n* = 6). Data are presented as mean ± SD (ns), unpaired t‐test (*n* = 6). (H) Representative image of *Cdkl5*‐WT and KO hippocampal neurons (DIV4) stained for tyrosinated tubulin (Tyr) in magenta and Detyrosinated tubulin (GLU) in green. Scale bar 20 μm. Image orientation has been adjusted to facilitate direct comparison between the staining of WT and KO growth cones. (I) Graph showing the quantification of the Tyr/GLU gradient in WT and KO neurons analyzed as in D. As shown no differences were observed between the two samples. Data are presented as mean ± SD (ns), multiple t‐test (67 *Cdkl5*‐WT and 73 *Cdkl5‐*KO neurons were analyzed).

We recently demonstrated that CLIP170 is mislocalized with respect to MTs in the growth cone of *Cdkl5*‐KO neurons [3]; we thus wondered whether the absence of CDKL5 might have a similar effect on the distribution of p150^glued^ in neuronal growth cones. To address this, we stained *Cdkl5*‐WT and ‐KO neurons (DIV4) for p150^glued^ and tubulin as a marker of neuronal morphology, considering the longest neurite as the axon; the fluorescence intensity ratio between p150^glued^ and tubulin was quantified along a line of 40 μm that comprised the axonal growth cone (Fig. 4A–C). As already demonstrated in the literature, WT neurons p150^glued^ presented a spatial gradient of intensity, which stretched ~10 μm from the axonal tip and then decreased moving proximally toward the cell body [26]. Interestingly, *Cdkl5*‐KO neurons were not characterized by the distal accumulation of the protein that presented a more linear intensity pattern (Fig. 4D). This difference was not due to a reduction of p150^glued^ expression level in *Cdkl5*‐KO neurons with respect to WT neurons, as shown in Fig. 4F,G. The same neurons were further analyzed for the distribution of MTs in the axonal growth cones. In line with our previous publication, the area occupied by alpha‐tubulin within a fixed area of the growth cones was significantly increased in *Cdkl5*‐KO neurons with respect to the WT counterpart (Fig. 4E).

Importantly, p150^glued^ preferentially localizes to the distal part of the neuronal axon, a region particularly enriched for tyrosinated (Tyr) alpha‐tubulin (Tyr) MTs [27, 28]. Thus, to understand if the mis‐localization of p150^glued^ in *Cdkl5*‐KO neurons might be caused by altered tyrosination of MTs, we analyzed the pattern of tubulin tyrosination along the distal axon, which is calculated by the ratio between tyrosination/detyrosination (Tyr/GLU). In line with the literature [29], we found that in *Cdkl5*‐WT neurons the signal presented a peak starting from the tip of the axon and stretching for ~20 μm and then decreased moving proximally toward the cell body (Fig. 4H); this gradient was observed also in *Cdkl5*‐KO neurons, indicating that tyrosination of microtubules is unaltered in the absence of Cdkl5 (Fig. 4I). This suggests that CDKL5 controls the correct localization of p150^Glued^ on neuronal MTs, which does not depend on tubulin tyrosination.

### CDKL5 is required for axonal bidirectional transport and efficient initiation of transport from the distal axon

**Fig. 5:**
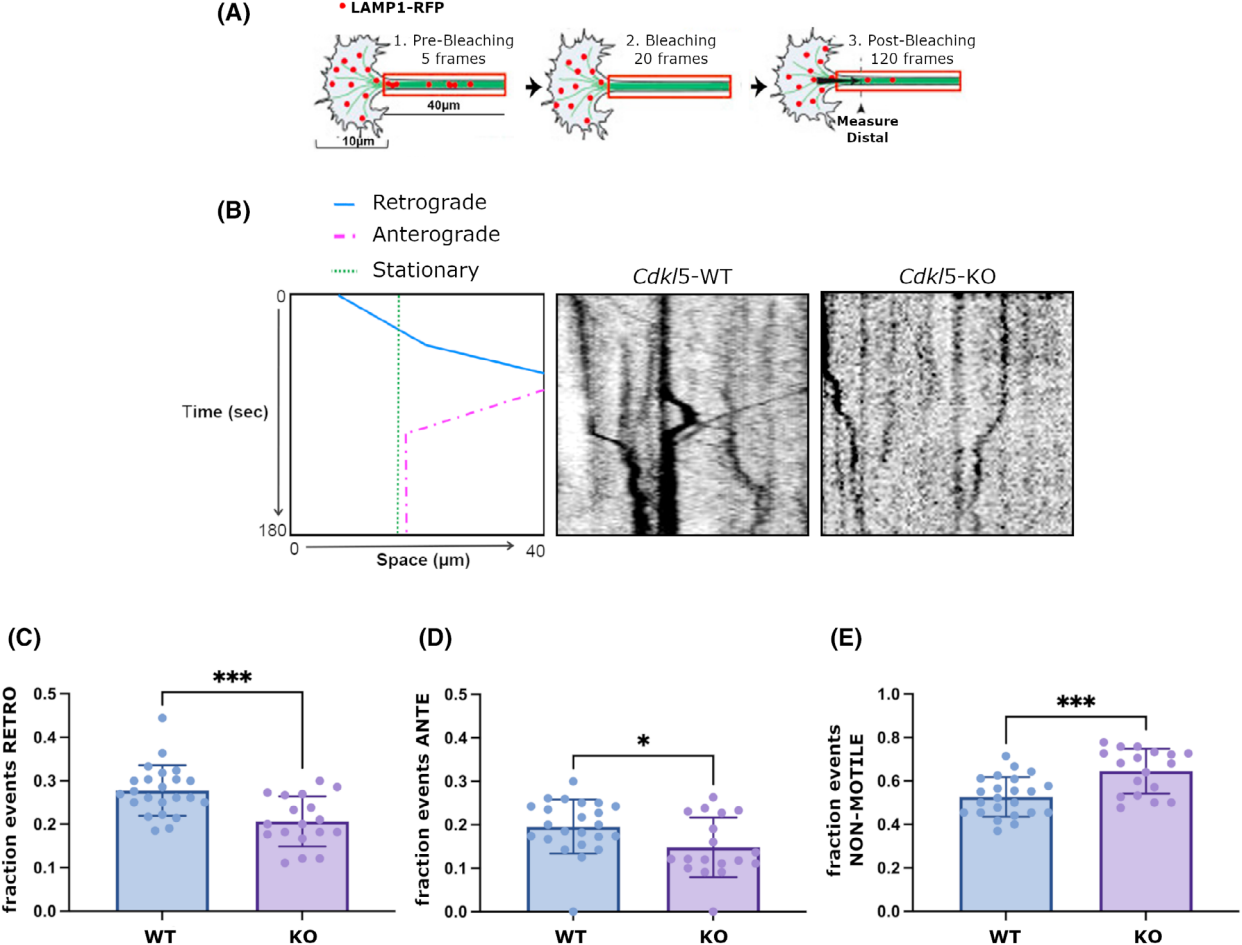
CDKL5 is required for axonal bidirectional transport and efficient initiation of transport from the distal axon. (A) Schematic image of live‐cell imaging of retrograde transport initiation. A 40 μm axonal region (10 μm proximal to the distal growth cone tip) was bleached, and the flux of LAMP‐1‐RFP organelles moving in the retrograde direction from the growth cone into the bleached region was measured in *Cdkl5*‐wild‐type (WT) and knockout (KO) hippocampal neurons at days *in vitro* (DIV) 3–4 through fluorescent recovery after photobleaching (FRAP). (B) Representative kymographs for *Cdkl5‐*WT and KO hippocampal neurons at DIV 3–4 with a schematic tracing of the analyzed vesicles. In the kymograph, the *x*‐axis represents the distance traveled (in μm), and the *y*‐axis represents time (in seconds). (C–E) The histograms show the mean ± SD of fraction events for retrograde (C), anterograde (D), and not‐motile (E) movements on the total amount of vesicles analyzed (23 WT vs 18 KO from at 3 different neuronal preparations). Data are presented as mean ± SD, unpaired *t*‐test (*P < 0.05, ***P < 0.001).

As mentioned, CLIP170 was previously demonstrated to be required for the correct recruitment of dynactin at the MT‐tips not only in proliferating cells but also in the distal axon of neurons where it is needed for efficient initiation of retrograde axonal transport [19]. Considering that both CLIP170 and dynactin are mislocalized from the axonal tip of *Cdkl5*‐KO neurons, we hypothesized that the absence of CDKL5 might impact the initiation of retrograde axonal transport. To test this hypothesis, we overexpressed LAMP1‐RFP to mark late endosomes/lysosomes and used a photobleaching assay to assess the number of cargos that initiate retrograde transport from the distal axon [19]. Photobleaching all fluorescently labeled cargos in a region‐of‐interest just proximal to the end of the axon allowed us to use live‐cell imaging to measure more accurately the number of cargos that entered the bleached region (Fig. 5A–C) without interference from the cargo already moving along the axon.

Measurements of cargo flux from the distal axon revealed a significant decrease in the number of LAMP1‐positive cargos moving retrogradely in *Cdkl5*‐KO with respect to WT neurons (Fig. 5D). Considering that LAMP1 organelles were motile not only in retrograde but also in anterograde direction, we also quantified the number of anterograde and nonmotile cargos; of note, in *Cdkl5*‐KO neurons we found a significant reduction in the number of anterograde cargos and an increase in the number of nonmotile ones (Fig. 5E,F). These results indicate that CDKL5 is required not only for the initiation of retrograde transport but also for anterograde motility along the axon.

### Treatment with pregnenolone restores CLIP170‐p150^glued^ interaction and axonal transport in *Cdkl5*‐KO neurons

**Fig. 6:**
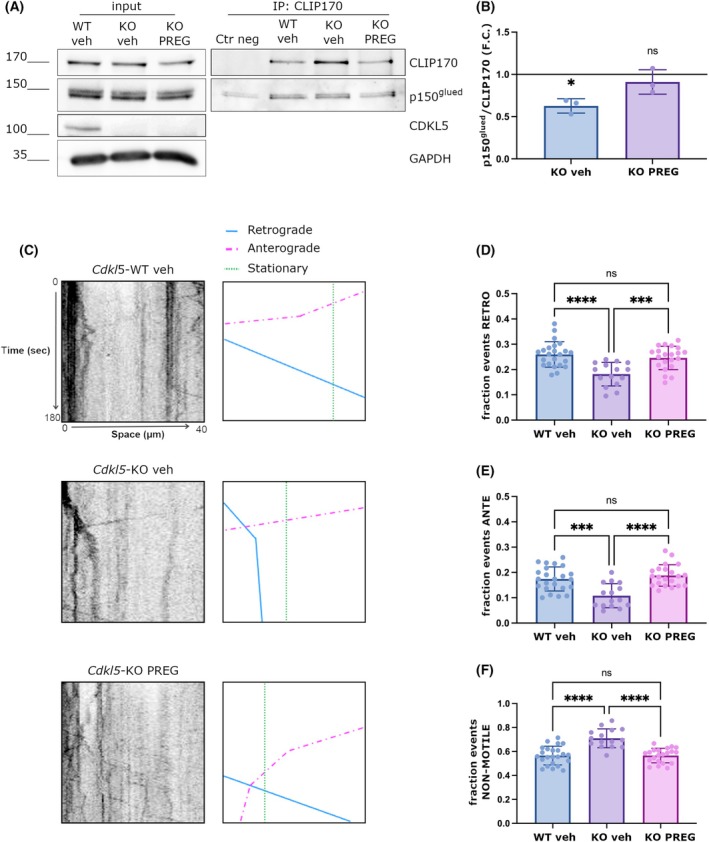
Treatment with pregnenolone (PREG) restores CLIP170‐p150^glued^ interaction and axonal transport in *Cdkl5*‐knockout (KO) neurons. (A) CLIP170 was immunoprecipitated from whole‐cell lysates from *Cdkl5*‐wild‐type (WT) and knockout (KO) neurons at DIV 7, treated with vehicle or PREG; IgGs were used as a negative control. Immunoprecipitates were analyzed by western blotting with CLIP170 antibody (IP) and p150^glued^ antibody (CoIP). (B) Graph showing the relative amount of p150^glued^ that co‐precipitates with CLIP170 as a fold change (F.C.) of the WT condition. Data are presented as mean ± SD (*P < 0.05), one sample *t*‐test (*n* = 3). (C) Representative kymographs of *Cdkl5*‐WT and KO vehicle and *Cdkl5*‐KO PREG treated neurons at DIV 3/4 with a schematic tracing of the analyzed vesicles. In the kymograph, the x‐axis represents the distance traveled (in μm), and the *y*‐axis represents time (in seconds). (D–F) Graph showing respectively the fraction of retrograde (D), anterograde (E) and nonmotile (F) transport events in *Cdkl5*‐WT and KO vehicle and *Cdkl5*‐KO pregnenolone (PREG) treated neurons. Data are shown as mean ± SD (***P < 0.001, ****P < 0.0001), one‐way ANOVA, Tukey *post hoc* test (23 *Cdkl5*‐WT vehicle, 15 *Cdkl5*‐KO vehicle and 21 *Cdkl5*‐KO PREG neurons were analyzed).

As mentioned, CLIP170 is the endogenous receptor of the neuroactive steroid pregnenolone (PREG) which, by promoting its open‐functional conformation, increases CLIP170 affinity for molecular partners such as p150^glued^ [16]. In proliferating cells silenced for CDKL5, the CLIP170‐p150^glued^ interaction is significantly reduced (Fig. 6A). We proceeded to analyze whether PREG might restore the capacity of CLIP170 to associate with p150^glued^ in *Cdkl5*‐KO neurons. *Cdkl5* WT and KO neurons were therefore treated at DIV1 with vehicle or PREG and CLIP170 was immunoprecipitated from lysates collected at DIV7. We analyzed the amount of CLIP170 and p150^glued^ by WB and, as shown in Fig. 6A, CLIP170 was efficiently immunoprecipitated in all conditions. Conversely, while the amount of p150^glued^ that coprecipitated with CLIP170 was significantly reduced by approximately 40% in the KO sample, the treatment with PREG restored the interaction (Fig. 6B), thus confirming its positive action on CLIP170 functionality. Considering the beneficial effect of PREG on the CLIP170‐p150^glued^ interaction, we decided to evaluate if the same treatment could ameliorate the initiation of retrograde transport in KO neurons. To this aim, we treated *Cdkl5*‐WT and KO neurons expressing LAMP1‐RFP with vehicle and PREG for 72 h and analyzed the efficiency of retrograde transport through FRAP at DIV4 as above. As expected, the number of LAMP1‐positive cargos was significantly reduced in *Cdkl5*‐KO neurons but was restored to levels of WT neurons upon PREG treatment (Fig. 6C,D). The same treatment also ameliorates the motility of anterograde cargos and reduces the number of nonmotile ones (Fig. 6E,F). This result indicates that PREG can bypass the absence of CDKL5 promoting bidirectional axonal transport.

## Discussion

One of the primary functions of MTs in all cells is to act as railways for the transport of building blocks throughout the cell. MT‐associated proteins (MAPs) and MT PTMs mark the tracks for the recruitment of motor–cargo complexes that move along MTs in dendrites and polarized axons following a precise wave of flux defined as “retrograde,” from plus‐ to minus‐ends and “anterograde” in the opposite direction. Dynein and kinesins are the main motor proteins, the functions of which are strictly related to motor adaptors (such as dynactin) and MAPs [30].

*In vitro* and *in vivo* studies demonstrate that CDKL5 controls neuronal maturation partially through the interaction with MT‐binding proteins such as MAP1S and the +TIPs EB2 and CLIP170 [8]. The latter, in particular, has been demonstrated to play an important role in the initiation of retrograde axonal transport that is mediated by the dynein‐dynactin complex. Indeed, thanks to its ability to bind MT plus‐ends, CLIP170 contributes to extending the radius of MT search in the axonal growth cone, creating the docking site for the recruitment of dynactin. This in turn binds dynein leading, in the end, to the formation of a functional motor complex [29]. Loss of function mutation in CLIP170 impairs its correct binding to MTs and causes a depauperation of dynactin from the MT‐tips [31].

Here, we show that CDKL5 allows the interaction between CLIP170 and dynactin and promotes the correct localization of dynactin at the MT‐tips; indeed, the silencing of CDKL5 in both HeLa and COS7 cells with two different siRNAs alters the distribution of dynactin without affecting the expression levels of the protein. Importantly, the re‐expression of CDKL5 in the silenced cells restored the correct localization of dynactin at the MT‐tips, strongly emphasizing the direct involvement of CDKL5 in this process. Importantly, the same result was obtained upon the overexpression of a CDKL5 kinase‐dead derivative, thus suggesting that CDKL5 may represent a sort of hub that promotes the interaction between p150^Glued^ and CLIP170 independently from CDKL5‐associated phosphorylation events.

Our analysis further revealed a delocalization of dynactin in the distal axon of *Cdkl5*‐KO neurons, which, as we previously demonstrated [3], is further characterized by an enlargement in the area occupied by MTs. It is noteworthy that an alteration in the correct functioning of dynactin has been associated with morphological defects of the growth cone and with an improper formation of tight microtubule bundles in the axon, especially in the distal region, thus corroborating a functional cross‐talk between CDKL5 and dynactin‐associated transport machinery [32].

The enrichment of dynactin and CLIP170 in the neuronal distal axon promotes the flux of cargos toward the soma [26]. Accordingly, the number of retrograde movements in *Cdkl5*‐KO neurons was strongly reduced. This is in line with previous publications in which the silencing of the p150^Glued^ dynactin subunit was found to affect the retrograde flux of cargo from the distal axon [19]. Besides the defective retrograde transport, we also observed a reduction in the anterograde flux. This is not a surprise; indeed, it has been already demonstrated that the inhibition of the retrograde axonal transport machinery often results in a bidirectional impairment of cargo flux [19, 33, 34]. However, we cannot exclude that such a defect may be a direct effect of CDKL5 on anterograde transport. In line with this, Baltussen and colleagues recently demonstrated an involvement of CDKL5 in the regulation of anterograde transport in the dendrites of primary cortical neurons [35]. Even if a precise molecular mechanism at the basis of such a defect was not elucidated, the identification of kinesin 4A as a putative substrate of CDKL5 [36] opens interesting perspectives. In the same work, the authors observed only a tendency of an impairment in retrograde transport [35]. The different neuronal types, the subcellular compartment, and the stage of neuronal development used in that study may explain the difference with our results.

Importantly, the observed defects in dynactin localization are further supported by CLIP170 hypofunctionality. Indeed, as mentioned, it has been largely demonstrated that CLIP170 regulates the correct localization of dynactin at the MT‐tips [15]. Here, we demonstrated that the absence of CDKL5 in COS7 cells negatively affected the colocalization of dynactin and CLIP170 and caused an overall reduction in the length of both dynactin and CLIP170 comet‐like structures, which, in line with our previous results [13], indicates an impairment in MT affinity. Importantly, the binding of CLIP170 to MTs is also influenced, besides the conformational state of the protein, by the tubulin PTMs. In particular, tyrosinated tubulin, which is predominant in the axonal growth cone, was found to improve CLIP170 binding [29]. In *Cdkl5*‐KO neurons we did not observe any difference in the gradient of tyrosinated tubulin with respect to WT neurons, thus indicating that the CDKL5‐dependent hypofunctionality of CLIP170 is not due to a difference in tubulin tyrosination but to the intrinsic properties of the protein. In line with this speculation, we found that the treatment with PREG was able to restore the interaction between CLIP170‐dynactin and the efficiency of retrograde transport in *Cdkl5*‐KO neurons, thus corroborating the tendency of CLIP170 to be present in a closed‐inactive conformation when CDKL5 is missing. Of note, the same treatment had a beneficial effect on anterograde transport. To date, we are unable to define the molecular mechanism underlying PREG‐dependent enhancement of anterograde transport; however, we can hypothesize that by enhancing MT dynamics via CLIP170 [16], PREG could have a positive effect on overall neuronal trafficking. The conformational state of CLIP170 is also regulated by phosphorylation [15, 37, 38], but it is not yet known whether CDKL5 takes part in this process. However, the CDKL5 consensus sequence (RPXS) is not present in CLIP170, suggesting a mechanism based on the molecular interaction of the two proteins rather than on direct phosphorylation events.

In conclusion, with this preliminary work, we have demonstrated a possible link between CDKL5 and the regulation of retrograde transport. Indeed, it is reasonable to envisage that CDKL5, by regulating the conformation/functionality of CLIP170, is able to mediate the binding of dynactin at the MT plus‐ends. Dynactin, in turn, can complex with dynein and thereby function as an adaptor for the loading of cargoes and increasing the motor efficiency of dynein during transport initiation [39].

Axonal transport is crucial for neuronal functions and survival; in fact, when the transport machinery is defective, MT‐based transport of building blocks for neuronal formation and maintenance is disrupted, leading to an impairment of neuronal homeostasis and to some neurodegenerative and neurodevelopmental defects [40].

Considering CDD, we speculate that altered CLIP170‐dynactin functions may alter axonal transport leading to a reduction in neuronal survival. While the link between the loss of CDKL5 and neuronal death was recently demonstrated [20], it remains unknown whether an analogous neuronal degeneration is present in CDD patients. In this regard, recent studies have revealed that CDD patients suffer from brain deterioration [41, 42, 43]; thus, we cannot exclude that defective retrograde transport may be implicated in the pathogenesis of CDD. Further studies of CDD‐derived neurons are mandatory to address this speculation, leading to a more detailed understanding of the disease and the design of therapeutic interventions.

## Materials and methods

### Antibodies

For western blot and immunofluorescence experiments, we used primary and secondary antibodies as indicated in Table 1.

**Table 1:** Source and dilution of antibodies used.

Target	Host species	Application	Dilution	Catalog #	Company
Alexa Fluor anti‐chicken 488	goat	IF	1 : 1000	A‐11039	Invitrogen
Alexa Fluor anti‐mouse 555	goat	IF	1 : 1000	AB150114	Abcam
Alexa Fluor anti‐rabbit 488	goat	IF	1 : 1000	AB150077	Abcam
Alexa Fluor anti‐rabbit 405	goat	IF	1 : 1000	AB175674	Abcam
Alexa Fluor anti‐rat 647	goat	IF	1 : 1000	AB150159	Abcam
CDKL5	rabbit	WB	1 : 1000	12973‐1‐AP	Proteintech
		IF	1 : 100		
CDKL5	mouse	WB	1 : 1000	sc‐376314	Santa Cruz Biotechnology
CLIP170	rabbit	WB	1 : 1000	GTX117504	Genetex
GAPDH	rabbit	WB	1 : 2000	G9545	Sigma‐Aldrich
GFP	chicken	IF	1 : 100	AB16901	Millipore
p150	mouse	WB	1 : 1000	610473	BD Bioscience
		IF	1 : 100		
αTubulin	rat	IF	1 : 1000	AB6160	Abcam
αTubulin tyrosinated	mouse	IF	1 : 1000	T9028	Sigma‐Aldrich
αTubulin detyrosinated	rabbit	IF	1 : 1000	AB48389	Abcam

### Plasmids

The following plasmids have been used for cell transfection: mEmerald‐CLIP170‐N‐18 (plasmid #54044; Addgene, Watertown, MA, USA), pCMV‐flag2B, pCMV‐flag2B‐mCDKL5, pCMV‐flag2B‐mCDKL5‐siCDKL5#1 resistant (flag‐CDKL5*), and pCMV‐flag2B‐mCDKL5‐siCDKL5#1 resistant A40V (flag‐CDKL5* A40V) (obtained by mutagenesis using the NEB Q5 site‐directed mutagenesis kit, E0554S), Lamp1‐RFP (plasmid #1817; Addgene).

### Cell cultures

COS‐7 (RRID:CVCL_0224) and HeLa (RRID:CVCL_0030) cell lines were obtained from ATCC. Cells were cultured according to standard protocols recommended by the supplier. The cell lines were not authenticated in our laboratory; however, they were routinely tested for mycoplasma contamination using a nested PCR‐based method, as described by Tang *et al*. [44]. Only mycoplasma‐free cultures were used in all experiments.

Cells were cultured in Dulbecco's modified Eagle's medium (DMEM, ECM0101L; EuroClone) supplemented with 10% fetal bovine serum (FBS, ECS0180L; EuroClone), L‐glutamine (2 mm, ECB3000D; EuroClone, Charleston, SC, USA), and penicillin/streptomycin (100 units per mL and 100 μg·mL^−1^ respectively, ECB3001D; EuroClone) at 37 °C with 5% CO_2_.

### Mouse Colony and maintenance

Cdkl5‐knockout (KO) mice on a CD1 genetic background, as previously described by Amendola *et al*. [7], were used in this study and were generously provided by Prof. Maurizio Giustetto. The day of birth was designated as postnatal day 0 (PND 0). After weaning, three to five littermates were housed together in enriched cages under a 12‐h light/dark cycle, in a temperature‐ and humidity‐controlled environment with *ad libitum* access to food and water. Animals were monitored daily to assess their general health status. Genotyping was performed by PCR on genomic DNA extracted from tail biopsies. Two forward primers were used: 5′‐ACGATAGAAATAGAGGATCAACCC for identification of the null allele, and 5′‐CCCAAGTATACCCCTTTCCA for the wild‐type (WT) allele. A common reverse primer was used: 5′‐CTGTGACTAGGGGCTAGAGA. The resulting PCR products yielded a single 340‐bp band for homozygous KO animals, a single 240 bp band for WT animals, and both bands for heterozygous females.

Protocols and use of animals were approved by the Animal Ethics Committee of the University of Insubria and in accordance with the guidelines released by the Italian Ministry of Health (D.L. 2014/26, license number 198/2018‐PR) and the European Community directives regulating animal research (2010/63/EU).

Adult mice were euthanized by cervical dislocation, while neonates were sacrificed by decapitation.

### Primary hippocampal neurons and pharmacological treatment

Primary hippocampal neurons were obtained as described in Barbiero *et al*. 2020 [3]. Briefly, neurons were prepared from E17 *Cdkl5*‐WT and KO mouse embryos obtained by crossing heterozygous females with WT males. After genotyping, hippocampi were isolated, enzymatically dissociated, and plated at high density (60 000 cells per well for p150glued analysis) or at low density (7500 cells per well for western blot and PTMs analysis). Neurons were cultured in Neurobasal medium and treated with cytosine arabinoside at DIV3 to limit glial proliferation. Pregnenolone (PREG; Sigma‐Aldrich, St. Louis, MO, USA) was dissolved in 100% EtOH and used at a final concentration of 1 μm (3). EtOH was used at a final concentration of 0.0032%. Neurons were treated with PREG or EtOH at DIV1 and analyzed at DIV4.

### Cell silencing and transfection

For silencing experiments, COS7 or HeLa cells were cultured in 24‐well plates and transfected with 20 nm siRNA oligonucleotides targeting CDKL5 (siCDKL5#1 5′CUAUGGAGUUGUACUUAAAUU3′; siCDKL5#2 5′GCAGAGUCGGCACAGCUAUUU3′) or a control siRNA siCtr 5′CGUACGCGGAAUACUUCGATT3′ using Lipofectamine™ RNAiMAX (13 778 075; Life Technologies Incorporated, Carlsbad, CA, USA).

For plasmid transfection, Lipofectamine™ 3000 or 2000 (L3000‐008 or 11 668 019, Life Technologies Incorporated) were used for cells and neurons respectively.

### Immunofluorescence

Neurons were fixed with a 4% formaldehyde (FA) solution prepared with 16% formaldehyde (28 908; Thermo Fisher Scientific, Waltham, MA, USA) supplemented with 4% sucrose (S7903; Sigma‐Aldrich) in Tris‐Buffered Saline (TBS, 20 mm Tris HCl pH 7.4, 150 mm NaCl (31 434; Sigma‐Aldrich)).

COS7 and HeLa cells were fixed 5 min in ice‐cold 100% methanol following 10 min in 4% FA supplemented with 4% sucrose (prepared as above described).

In both cases, cells were blocked for 1 h (or 24 h for rescue experiments) at room temperature in a permeabilizing blocking solution (5% goat serum ECS0200D; Euroclone), 0.2% Triton X‐100 (T8787‐250ML; Sigma‐Aldrich), PBS 1× (ECB4004L; EuroClone) before incubation with the appropriate primary antibodies (overnight at 4 °C); subsequently, secondary antibodies were applied (1 h at RT). Both incubations were performed in blocking solution.

### p150^glued^–comet quantification

Images of Hela cells stained for p150^glued^, tubulin, and CDKL5 were acquired using the.

LEICA TCS SL confocal microscope (LEITZ; Leica Mycrosystems, Wetzlar, Germany) with objective 63× (NA 1.32; zoom factor = 8) and the pinhole set at 1 Airy unit.

For each cell analyzed, we selected a ROI (10μm^2^) at the periphery of the cell starting from the end of MTs, and we measured with the ImageJ software the length of p150^glued^ comet‐like structures. In accordance with the literature [22] the comets whose length was greater than 1.5 μm were included in the analysis. We performed four different experiments in which we quantified 237 Ctr cells, 261 si*CDKL5*#1 cells, and 117 siCDKL5 #2 cells, respectively. For each cell, we quantified the percentage of comets greater than 1.5 μm and we plotted the mean of this percentage for each experiment.

For the rescue experiment, an intensity threshold was set with the ImageJ software to identify CDKL5‐expressing cells from CDKL5‐silenced ones stained with CDKL5 antibody (12973‐1‐AP; Proteintech, Rosemont, IL, USA). The same threshold was applied for all the quantified images. The percentage of p150^glued^‐comet positive or negative cells was measured as mentioned before (109 and 100 cells for siCtr and siCDKL5#1 transfected with the empty pCMV vector respectively, and 66 and 69 siCDKL5#1 cells transfected with flag‐CDKL5* or flag‐CDKL5* A40V vector respectively; *n* = 3).

### Comet length and colocalization analysis

Images of COS7 cells stained for dynactin in magenta, CLIP170‐GFP in green, and tubulin in cyan were acquired with a LEICA TCS SL confocal microscope (LEITZ; Leica Mycrosystems, Wetzlar, Germany) with an objective 63× (NA 1.32; zoom factor = 8) and the pinhole set at 1 Airy unit. Using the ImageJ software, a threshold was set for dynactin (40–255) to reduce the non‐specific signals.

Two ROI of 15 μm^2^ were further identified at the periphery of each cell, within which we selected MTs (tubulin‐positive structures), and we quantified the length of CLIP170 and dynactin accumulation (comet‐like structures) on selected MTs. CLIP170 positive tips were used to easily identify microtubule plus ends. To evaluate the degree of co‐localization between dynactin and CLIP170, Manders' coefficient was calculated.

### p150^glued^ analysis in neurons

High‐density neurons fixed at DIV 4 were stained for p150^glued^ (magenta) and tubulin (green) to easily identify neuronal morphology; the longest neurite was considered the axon. Images were acquired using the Olympus BX51 (Tokyo, Japan) fluorescent microscope with 60× objectives. The Ocular imaging software (QImaging) was used to import images from the camera (Retiga R1 CCD camera; QImaging, Surrey, BC, Canada).

Using the ImageJ software, a segmented line of 40 μm was drawn looking at the tubulin signal starting from the end of the growth cone, and a plot profile of p150^glued^ and tubulin was obtained. The accumulation of p150^glued^ at the growth cone was calculated as a ratio between the plot profile intensity signal of p150^glued^ and tubulin.

### FRAP analysis

We performed fluorescent recovery after photobleaching (FRAP) to assess the retrograde movement of LAMP‐1‐RFP–positive organelles in hippocampal neurons from Cdkl5‐WT and KO mice at DIV3/DIV4. A 40‐μm segment of the axon, located approximately 10 μm from the tip of the distal growth cone, was photobleached, and the influx of vesicles from the growth cone into this region was monitored. Live‐cell imaging was conducted using a Leica SMD SP8 confocal microscope (Leica Microsystems, Wetzlar, Germany) equipped with a 63× objective and LASX software, maintaining cells at 37 °C. Data acquisition was carried out in a blinded manner by an operator at the Advanced Light and Electron Microscopy BioImaging Center (ALEMBIC, Ospedale San Raffaele, Milan). Time‐lapse images were recorded at a rate of 1.3 frames per second, beginning 5 s before and continuing for 120 s after bleaching. Photobleaching was induced using a 561 nm laser at full power (100%) for 20 scan cycles.

Vesicle dynamics were analyzed using the Multi‐Kymograph plugin in ImageJ (BIII). Retrograde transport was defined as the movement of vesicles from the distal side of the axon into the bleached area by a minimum distance of 3.5 μm. The number of such vesicles was manually quantified for each neuron (23 WT and 18 KO neurons, from three independent neuronal cultures).

To evaluate overall cargo motility, all vesicle trajectories extracted from kymographs were categorized as anterograde, retrograde, or nonmotile. Vesicles showing a net displacement greater than 10 μm in one direction were classified as motile (anterograde or retrograde), whereas those with displacements less than 10 μm were considered nonmotile.

### PTMs analysis

A 50‐μm‐long line starting outside the growth cone and continuing along the axon was drawn for *Cdkl5*‐WT and KO hippocampal neurons at DIV4. Immunofluorescence staining was performed for tubulin tyrosination (Tyr) in red and de‐tyrosination (GLU) in green acquired with a fluorescence microscopy (Olympus BX51 (Tokyo, Japan) fluorescent microscope with 40× objectives). The line intensity profiles of the ratio Tyr/GLU were measured in the same distal axon for each neuron, using data from 67 *Cdkl5*‐WT and 73 KO neurons that were derived from six different neuronal preparations.

### Western blot and immunoprecipitation

Cells seeded in a 24‐well plate were collected in 110 μL of Laemmli buffer 3× (Laemmli buffer 6×: 0.3 M TRIS–HCl (pH 6.8), 10% SDS, 30% glycerol, 0.01% bromophenol blue, 12% beta Mercaptoethanol) after a rapid wash in PBS 1x, sonicated (3 cycles, 80 A, 5 pulses) and boiled (5 min, 95 °C). 30 μL of sample was loaded before being separated by either 8 or 10% SDS/PAGE, transferred to nitrocellulose membranes, and blocked in 5% nonfat milk in TBS 1x supplemented with 0.2% Tween‐20 (P7949; Sigma‐Aldrich) (TBS‐T). Blots were incubated with primary antibodies (1 : 1000 for all the antibodies except the GAPDH that we used 1 : 20000, prepared in milk) overnight at 4 °C, washed in TBS‐T, and incubated with appropriate secondary antibodies diluted 1 : 10000 (except for the GAPDH used 1 : 20000) in 5% non‐fat milk TBS‐T for 1 h at RT. After extensive washes, blots were developed with a chemiluminescence‐based detection system ECL (Genespin, Milan, Italy) coupled to G:BOX Chemi Imaging System (Syngene, Bangalore, India). Densitometric analyses of protein expression were performed using ImageJ Software. Protein expression levels were normalized using GAPDH as an internal standard. The amount of lysate was determined for each protein through linear range analysis procedures.

For IP experiments, hippocampal neurons or HeLa cells were lysed in lysis buffer (50 mm Tris‐ HCl pH 7.5, 150 mm NaCl, 1% Triton X‐100, 1 mm EDTA, 1 mm EGTA, protease inhibitor cocktail and PhosSTOP). 500 μg of lysates were incubated overnight at 4 °C with 0.5 μg of anti‐CLIP170 or unrelated IgGs as control. The immunocomplexes were precipitated with protein‐G Agarose (Life Technologies, Carlsbad, CA, USA), washed several times with lysis buffer, and analyzed by SDS/PAGE.

### Statistical analysis

Data quantifications were analyzed with the Prism software (graphpad) and all values are expressed as the mean±SD. The significance of results was evaluated by one‐way ANOVA followed by Dunnet's or Tukey's post hoc test, unpaired Student's *t*‐test, or one‐sample t‐test according to the type of experiment analyzed. Probability values of *P* < 0.05 were considered statistically significant.

## Author contributions

Conception and design; IB. Acquisition of data or analysis and interpretation of data; SB, CC, RDR, IB. Manuscript drafting or revising it critically for important intellectual content; SB, CC, IB, GV. Final approval of the version to be published; SB, CC, RDR, IB, GV. Agreed to be accountable for all aspects of the work in ensuring that questions related to the accuracy or integrity of any part of the work are appropriately investigated and resolved; SB, CC, IB, GV.

## Conflict of interest

The authors declare no conflict of interest.

## Data Availability

The data that support the findings of this study are available from the corresponding author.
